# HOX-mediated *LMO2* expression in embryonic mesoderm is recapitulated in acute leukaemias

**DOI:** 10.1038/onc.2013.175

**Published:** 2013-05-27

**Authors:** F J Calero-Nieto, A Joshi, N Bonadies, S Kinston, W-I Chan, E Gudgin, C Pridans, J-R Landry, J Kikuchi, B J Huntly, B Gottgens

**Affiliations:** 1 Department of Haematology, Wellcome Trust and MRC Cambridge Stem Cell Institute, Cambridge Institute for Medical Research, Cambridge University, Cambridge, UK; 2 Division of Stem Cell Regulation, Center for Molecular Medicine, Jichi Medical School, Shimotsuke, Tochigi, Japan

**Keywords:** gene regulation, haematopoiesis, HOX, leukaemia, LMO2

## Abstract

The Lim Domain Only 2 (*LMO2*) leukaemia oncogene encodes an LIM domain transcriptional cofactor required for early haematopoiesis. During embryogenesis, *LMO2* is also expressed in developing tail and limb buds, an expression pattern we now show to be recapitulated in transgenic mice by an enhancer in *LMO2* intron 4. Limb bud expression depended on a cluster of HOX binding sites, while posterior tail expression required the HOX sites and two E-boxes. Given the importance of both *LMO2* and HOX genes in acute leukaemias, we further demonstrated that the regulatory hierarchy of HOX control of *LMO2* is activated in leukaemia mouse models as well as in patient samples. Moreover, *Lmo2* knock-down impaired the growth of leukaemic cells, and high *LMO2* expression at diagnosis correlated with poor survival in cytogenetically normal AML patients. Taken together, these results establish a regulatory hierarchy of HOX control of *LMO2* in normal development, which can be resurrected during leukaemia development. Redeployment of embryonic regulatory hierarchies in an aberrant context is likely to be relevant in human pathologies beyond the specific example of ectopic activation of *LMO2*.

## Introduction

The Lim Domain Only 2 (*LMO2*) gene encodes a transcriptional cofactor that forms multiprotein complexes with other transcription factors, such as Ldb1, Scl/Tal1, E2A and Gata1/Gata2^1^ and is widely expressed within the haematopoietic system with the exception of T cells. Mice lacking *LMO2* die around embryonic day E10.5 because of a complete failure of erythropoiesis.^2^ *LMO2* is a major oncogene in T-cell acute lymphoblastic leukaemia (T-ALL) and was originally identified through its involvement in recurrent chromosomal translocations.^3, 4^ Importantly, transgenic mouse models confirmed that ectopic expression of *LMO2* in T cells constitutes an initiating leukaemogenic lesion.^5^ Three distinct promoters and eight enhancer elements dispersed over 100 kb have been identified in the regulation of the *LMO2* gene.^6, 7, 8, 9, 10^ By contrast, the regulatory pathways that direct abnormal expression in T-ALL patients without *LMO2* translocations are much less well understood. Since aberrant ectopic *LMO2* expression in T-ALL is much more prevalent than translocations involving the *LMO2* locus,^11^ dysregulation of transcriptional pathways upstream of *LMO2* needs to be recognized as a potentially leukaemogenic event.

Homeobox genes are essential developmental regulators. Their abnormal expression is frequently observed in lymphoid, myeloid and mixed-lineage leukaemias (MLL), and is characteristic of translocations involving the MLL gene.^12^ High-level expression of HOXA genes can also be seen in leukaemia patients without MLL translocations^13, 14^ and confers poor prognosis in both T-ALL and acute myeloid leukaemia (AML)^12, 15^ thus emphasizing the need for further research to identify key downstream mediators of the leukaemic phenotype.

Here, we report the characterization of an enhancer responsible for *Lmo2* expression in the embryonic tail bud and limbs. Using extensive transgenic mouse analysis, we demonstrate that this enhancer depends upon highly conserved HOX and E-box motifs. Moreover, the enhancer also functions in leukaemia cell lines, displays active histone marks in primary patient samples and can be activated by HOXA5. Finally, we provide evidence to suggest that elevated levels of *LMO2* contribute to the leukaemic phenotype in AML, beyond the conventional role of *LMO2* as a T-ALL oncogene.

## Results

### Lmo2 displays anterior/posterior-specified expression domains during early development

Analysis of *Lmo2* knock-in transgenic mice that contain an *LacZ* reporter gene inserted into the *Lmo2* locus^16^ revealed consistent expression at embryonic day E12.5 in vessels, brain, eyes, somites, fetal liver, Progress Zone (PZ) beneath the Apical Ectodermal Ridge region of limb buds, tail bud and developing limbs (Supplementary Figures 1i–iii). RNA *in situ* hybridization at E10.5 confirmed strong *Lmo2* expression in the PZ and posterior part of developing limbs as well as the tail bud (Supplementary Figures 1iv–v). By day E11.5, *Lmo2* expression in limb buds was largely confined to the PZ with only faint staining in the antero- and postero-distal regions. Tail bud staining was also weaker at E11.5 consistent with general downregulation of *Lmo2* in these early mesodermal tissues at midgestation. Taken together, therefore, RNA *in situ* hybridization and analysis of *Lmo2* knock-in embryos identified expression in limbs and tail bud, thus establishing non-haematopoietic expression domains of *Lmo2* that are organized in an anterior/posterior pattern, and therefore likely related to some of the major patterning activities during early development.

### A conserved enhancer in LMO2 intron 4 is active during early limb and tail bud development

**Figure 1:**
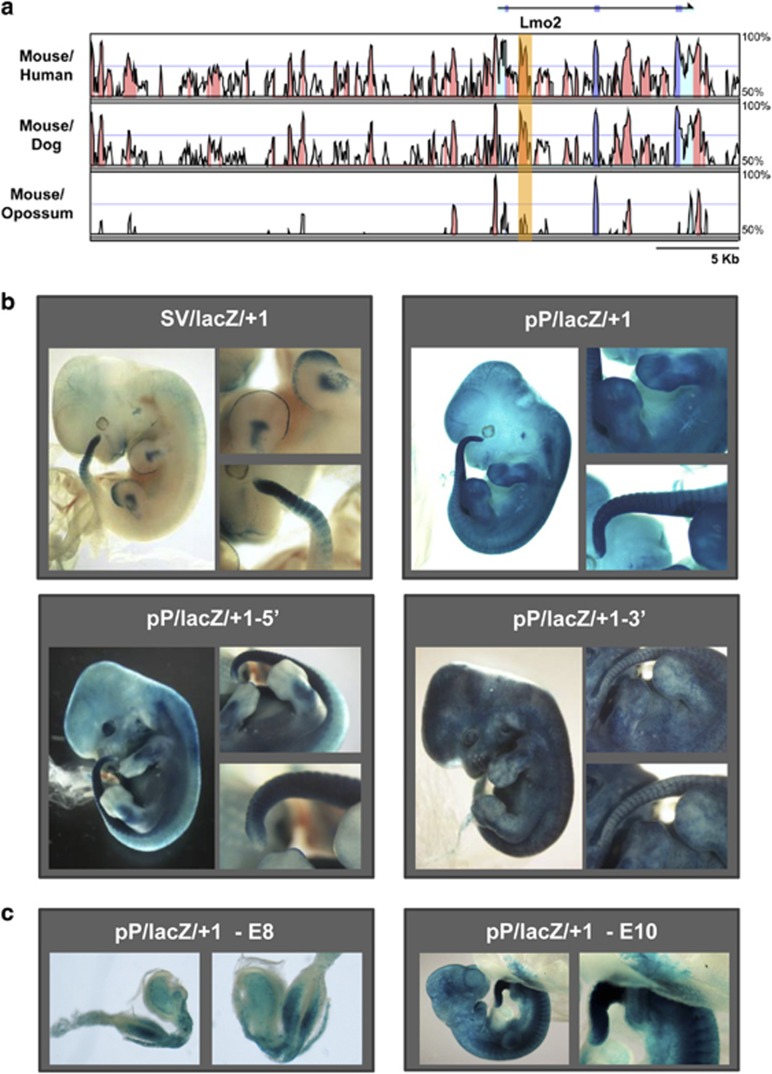
The *LMO2* +1 enhancer drives expression to the developing limb and tail buds as well as to endothelium in transgenic mouse embryos. (**a**) MVista representation of sequence conservation across the mouse *Lmo2* locus showing mouse/human, mouse/dog and mouse/opossum alignments. The conservation plots show regions with at least 50% of conservation (y axis). Peaks of sequence conservation in exons are shown in blue, those in transcribed but not in translated regions (3′ UTR and 5′ UTR) are shown in pale blue and those in non-coding regions are shown in orange. Arrows indicate the direction of transcription. The region corresponding to the +1 enhancer is highlighted. (**b**) Whole-mount staining of representative E12.5 transgenic mouse embryos for the constructs indicated, with close-up views of limbs and tail bud regions shown to the right of each whole-mount view. *LMO2* +1 enhancer directed expression of reporter gene to the PZ region, tail bud, developing limbs and endothelium with a heterologous SV40 (SV/lacZ/+1) and endogenous *LMO2* proximal promoter (pP/lacZ/+1). Transgenic embryos carrying 5′ region of +1 enhancer (pP/lacZ/+1-5′) presented staining in tail bud, developing limbs and endothelium. Transgenic embryos carrying 3′ region of +1 enhancer (pP/lacZ/+1-3′) only present strong endothelial staining. (**c**) Representative E8.5 and E10.5 mouse embryos from a transgenic mouse line carrying the pP/lacZ/+1 construct, showing staining in the PZ, developing limbs and tail bud area.

Our previous analysis of the *LMO2* locus identified a conserved region located 1 kb downstream of the *LMO2* ATG (hereafter referred to as +1 enhancer; Figure 1a), which directed expression to endothelial but not to blood cells when tested in transgenic mice in combination with the *LMO2* proximal promoter (pP).^8^ By contrast, the pP region alone only displayed weak endothelial activity.^9^ A more detailed analysis of transgenic embryos carrying the +1 enhancer demonstrated consistent activity in the PZ region, tail bud and developing limbs (Figure 1b). Importantly, this expression pattern was observed with both a heterologous SV40 and endogenous *LMO2* pP thus demonstrating classical enhancer activity.

**Figure 2:**
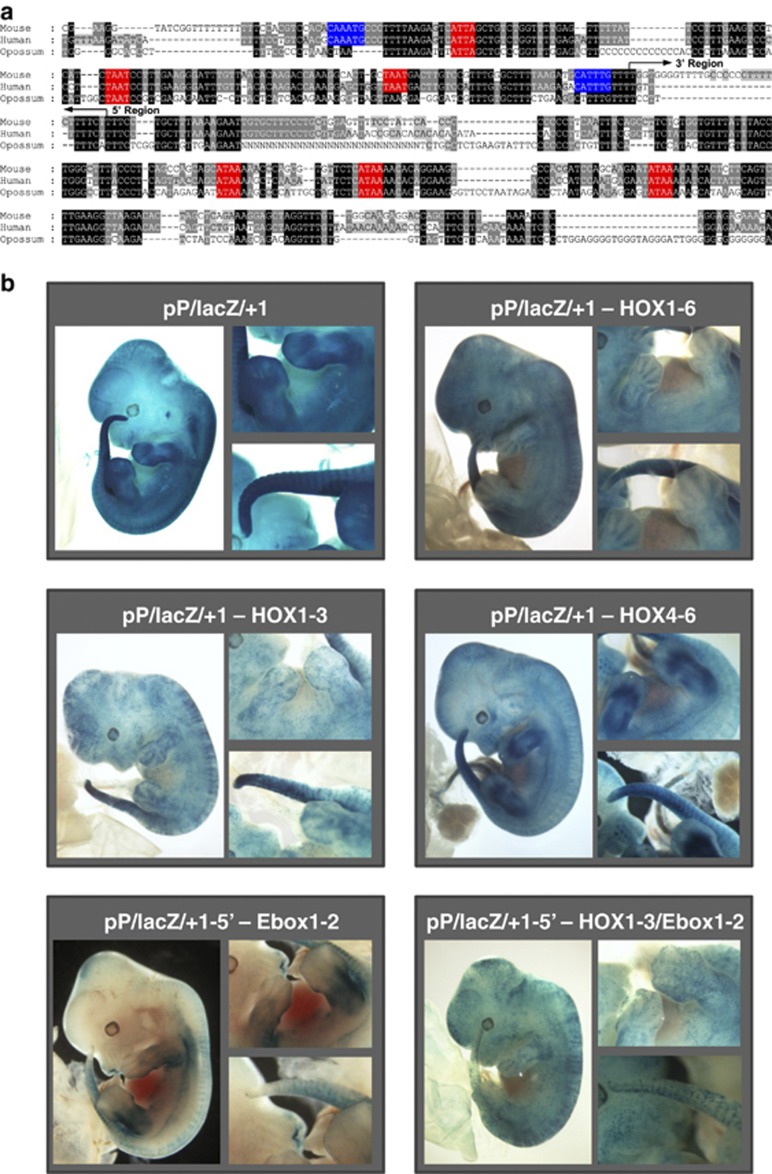
HOX and E-box motifs are critical for limb and tail bud activity of the +1 enhancer *in vivo*. (**a**) Nucleotide sequence alignment of the +1 enhancer with conserved HOX (red) and Ebox (blue) motifs marked. Arrows indicate the boundaries of the 5′ and 3′ deletion constructs. (**b**) Representative transgenic mouse embryos at E12.5 showing whole-mount X-Gal reporter expression driven by wild-type and mutated +1 enhancer constructs, with close-up views of limbs and tail bud regions shown to the right of each whole-mount view. Simultaneous mutation of all six putative HOX binding sites (pP/lacZ/+1–HOX1-6) or homeoboxes located in 5′ region of +1 enhancer (pP/lacZ/+1–HOX1-3) abolished staining in developing limbs and the PZ but did not affect endothelial or tail bud staining. Staining pattern of embryos carrying mutation of homeoboxes located in 3′ region of +1 enhancer (pP/lacZ/+1–HOX4-6) was indistinguishable from wild-type enhancer. Simultaneous mutation of two E-boxes located in 5′ region of +1 enhancer (pP/lacZ/+1-5′–Ebox1-2) caused a significant reduction in tail bud staining but did not affect staining in limbs and endothelium. Simultaneous mutation of three homeoboxes and two E-boxes located in 5′ region of +1 enhancer (pP/lacZ/+1-5′–HOX1-3/Ebox1-2) with only endothelial staining remaining.

Sequence analysis revealed two blocks of evolutionary conservation corresponding to the 5′ and 3′ regions of the element (Figure 2a). When the 5′ region was used to generate transgenic embryos, lacZ activity was retained in tail bud and developing limbs (Figure 1b). By contrast, staining in PZ, tail bud and developing limbs was abolished when using the 3′ region with only strong endothelial staining remaining. To define the activity of the +1 enhancer at multiple stages of early development, we generated a stable transgenic line containing the enhancer together with the pP. LacZ expression was observed as early as E8.5, with specific staining in PZ, developing limbs and tail bud area prominent by day E10.5 (Figure 1c). Taken together, these results suggest that the *LMO2* +1 enhancer has an important role in directing the expression of *Lmo2* to developing limbs and tail as well as to endothelial cells.

### Activity of the LMO2 +1 enhancer in the PZ and limb buds requires Homeobox consensus sites

Blocks of sequence conservation in the +1 enhancer (Figure 2a) included six putative consensus binding sites for homeodomain transcription factors (HOX boxes) as well as two putative E-boxes for basic Helix-Loop-Helix (bHLH) proteins. Of note, putative HOX binding sites located in the 5′ portion of the enhancer corresponded to a TAAT consensus, while those located in the 3′ portion matched an ATAA consensus. To assess the importance of the E-box and HOX consensus sites, mutant enhancer constructs were analysed in transgenic assays (Figure 2b). Simultaneous mutation of all six putative HOX binding sites did not affect endothelial expression or staining of tail bud, but abolished staining in developing limbs and PZ (Figure 2b). Mutation of the two E-boxes did not affect expression in limbs and endothelial cells, but caused a significant reduction in tail bud staining (Figure 2b).

Mutation of the three homeoboxes located in the 5′ region of the +1 enhancer (HOX1, HOX2 and HOX3; corresponding to TAAT consensus) resulted in a staining pattern similar to that seen with ablation of all six homeobox sites. On the other hand, when only homeoboxes located in the 3′ region were mutated (HOX4, HOX5 and HOX6; corresponding to ATAA consensus), the staining pattern was indistinguishable from wild-type enhancer. Interestingly, when HOX1, HOX2 and HOX3 motifs were mutated together with E-boxes 1 and 2, no staining of developing limbs, PZ or tail bud was observed with only endothelial staining remaining (Figure 2b). The staining pattern therefore was very similar to that obtained with only the 3′ part of the enhancer (see above and Figure 1b), thus suggesting that the HOX1, HOX2 and HOX3 motifs together with the two E-boxes have a critical role in the expression of *LMO2* in PZ, limb and tail bud. These results therefore suggest that expression of *Lmo2* during early limb and tail bud development is critically controlled by homeodomain and bHLH transcription factors.

### T-ALL patients with high HOXA gene expression show elevated levels of LMO2

**Figure 3:**
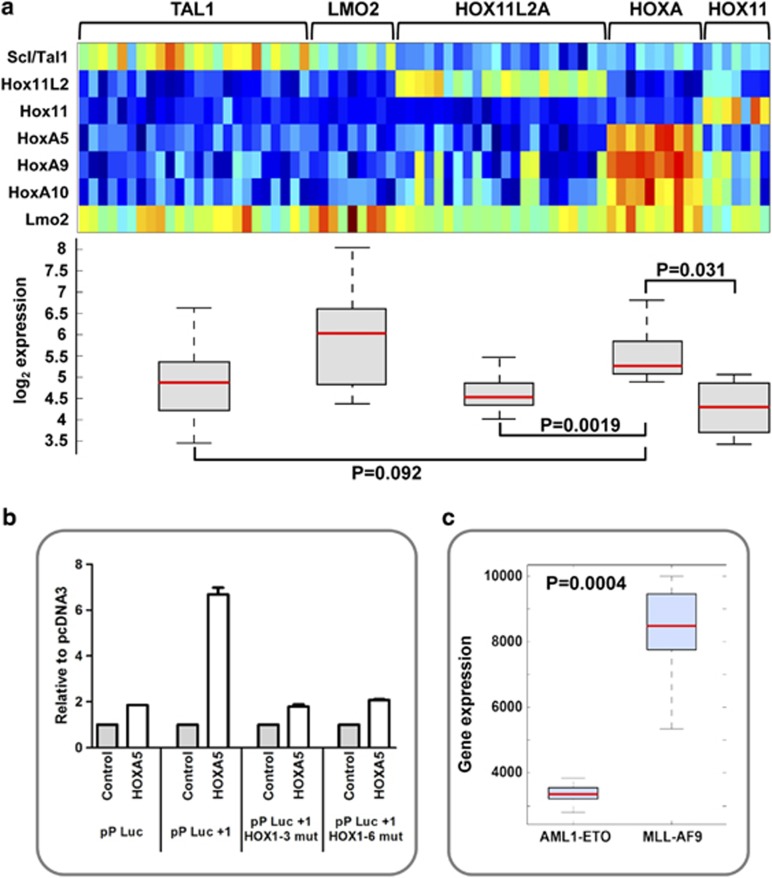
A link between HOX activation and the *LMO2* +1 enhancer in leukaemia cells. (**a**) Normalized gene expression data for 72 T-ALL patients having one of the major molecular cytogenetic abnormalities (TAL1, LMO2, HOXA, HOX11 and HOX11L2) shown by heatmap and boxplot. Elevated levels of *LMO2* are seen in both the LMO2 and HOXA categories. Corresponding *P*-values are shown. (**b**) HOXA5 can transactivate the *LMO2* +1 enhancer. Transient co-transfection assays in 293T cells show significant activation of the wild-type enhancer construct (pP/Luc/+1) but not the mutant enhancer with either first three or all HOX sites mutated (pP/Luc/+1HOX1-3mut and pP/Luc/+1HOX1-6mut, respectively). Values are expressed relative to the control pcDNA3, and the mean and s.e.m. for at least two independent transfections (each one performed in triplicate) are shown. (**c**) Normalized gene expression data for human CD34^+^ cells, obtained from cord blood, transformed with the leukaemia fusion genes MLL-AF9 (*n*=9) or AML1-ETO (*n*=6) shown by boxplot. Greater levels of *LMO2* can be observed in the MLL-AF9 category. Corresponding *P*-value is shown.

Translocations that place T-cell receptor enhancers in the vicinity of the *LMO2* gene represent one of the classical initiating mutations in T-ALL. Chromosomal rearrangements involving the *HOXA* cluster have also recently been identified in a subset of T-ALL patients.^13^ Following on from our discovery of a regulatory hierarchy between homeodomain factors and *Lmo2* during early embryonic development, we next investigated whether evidence for a similar link could be detected in T-ALL patients. To this end, we analysed *LMO2* expression in a published cohort of 67 T-ALL patients, grouped into 5 clusters largely based on their primary cytogenetic abnormality (TAL1, LMO2, HOX11, HOX11L2 and HOXA).^14^ *LMO2* expression was highest in those samples with *LMO2* and *TAL1* translocations, which are known to represent a T-ALL subgroup with similar genetic and phenotypic characteristics^13, 14, 17^ but was also elevated in patients with upregulation of *HOXA* locus genes (Figure 3a). Our identification of elevated *LMO2* levels in these patients suggests a link between *HOXA* genes and *LMO2*, likely to be relevant for T-ALL. Of note, the recent discovery of acquired mutations in *MEF2C* in a subset of immature T-ALL patients together with the observation that MEF2C can bind to the *LMO2* promoters has already established a precedent for LMO2 function as a potential leukaemia oncogene in this subset of ‘phenotypically early' T-ALL.^17^

### HOXA5 can transactivate the LMO2 +1 enhancer

T-ALL patients that correspond to the HOXA group are characterized by high levels of expression of *HOXA5*, *HOXA7* and *HOXA9* genes.^13, 14^ Staining of the central-posterior part of developing limbs observed in *LMO2* +1 enhancer transgenic embryos was similar to the expression pattern of central-posterior *HOXA* genes (including *HOXA5*, *HOXA7* and *HOXA9*) during early development. We therefore hypothesized that *LMO2* expression may be under direct control of *HOXA* genes. A recent study assessing the consensus binding sites for homeoproteins demonstrated that the core binding site for HoxA5 and HoxA7 is TAAT, while ATAA is the preferred consensus for HoxA9.^18^ Both motifs correspond to the conserved HOX sites present in the *LMO2* +1 region, with the HoxA5/HoxA7 TAAT motif corresponding to the functionally significant motifs present in the 5′ half of the enhancer.

We performed transactivation studies where constructs containing the LMO2 pP with and without the +1 enhancer were co-transfected with expression constructs for HOXA5, HOXA7 or HOXA9. Experiments were also performed using an HOX11 expression construct, since HOX11 represents a T-ALL oncogenic homeobox gene whose high expression constitutes an independent patient group without elevated *LMO2* expression (see Figure 3a).^13, 14, 19^ Transactivation assays performed with HOX11, HOXA7 or HOXA9 did not show significant enhancement of luciferase activity, even in the presence of their dimerization partner Meis1 (data not shown). By contrast, transactivations using HOXA5 resulted in ∼7-fold enhancement of luciferase activity, which was abolished following simultaneous mutation of the first three or all six conserved HOX motifs contained in the +1 enhancer (Figure 3b). Our data are therefore consistent with a model whereby *LMO2* is under the control of homeobox genes such as *HOXA5* acting through the +1 enhancer to mediate ectopic expression in a subset of T-ALL patients with immature phenotype.

### The LMO2 +1 enhancer carries active chromatin marks in AML patients

Immature T-ALL has long been recognized to share molecular and cellular features with AML, a notion underlined further by recent reports of genome sequencing in early precursor T-cell ALL (ETP-ALL).^20^ As shown above, *LMO2* expression in T-ALL correlates with upregulation of *HOXA* genes. Moreover, T-ALL samples with an HOXA signature and high LMO2 commonly display an immature ETP phenotype^13, 17, 19, 21^ and occasionally co-express both lymphoid and myeloid surface antigens.^13, 22^ However, T-ALL samples with translocations involving the *LMO2* locus generally present a more mature phenotype.^13, 17^ Moreover, the *HOXA*-inducing MLL or CALM-AF10 translocations are also often found in T-ALL patients with immature T-cell immunophenotypes,^13, 14, 22, 23^ thus suggesting a common theme of HOXA-LMO2 activation in this subgroup of T-ALL. Of note, both MLL and CALM-AF10 translocations are also found as recurrent translocations in AML,^24, 25^ and mouse models show both myeloid and lymphoid characteristics.^26^

**Figure 4:**
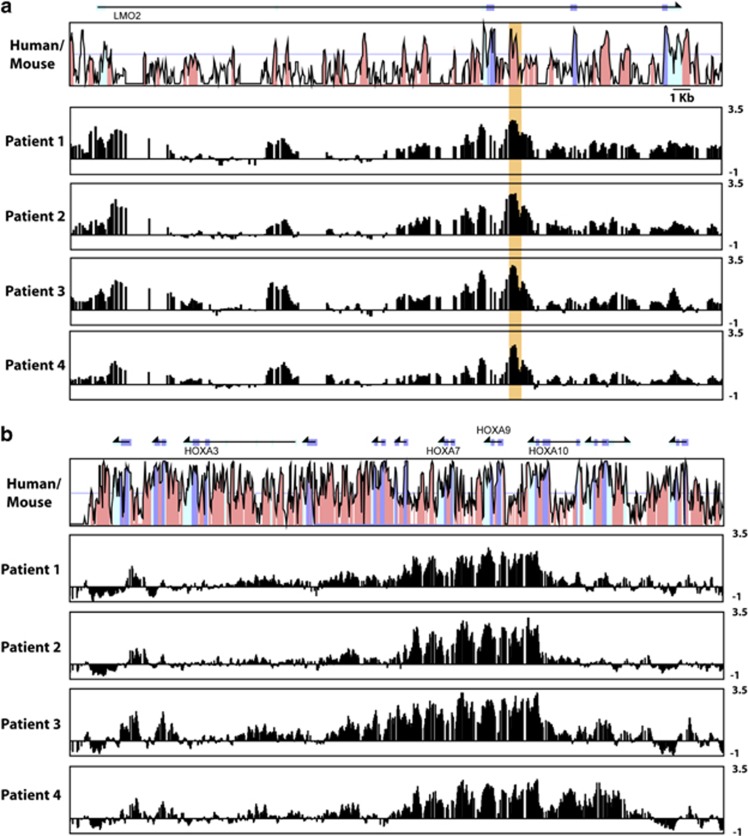
Acetylation of histone H3 lysine 9 indicates that the *LMO2* +1 enhancer is active in primary AML patient samples showing activation of the *HOXA* cluster. (**a**) ChIP-on-chip analysis of the human *LMO2* locus in four patient samples shows a peak of H3 lysine 9 acetylation at the *LMO2* +1 enhancer. MVista representation of human/mouse sequence conservation is shown at the top with the +1 enhancer highlighted. Annotations are as in Figure 2a. (**b**) ChIP-on-chip analysis of the *HOXA* cluster shows elevated H3 lysine 9 acetylation across central/posterior *HOXA* genes in the same four patient samples. Enrichment values are calculated as fold enrichment over the mean intensity across the whole locus and expressed as log base 2.

Compared with their relatively recent implication in T-ALL, HOXA genes have long been recognized as powerful mediators of AML. To investigate whether *HOXA* activation in AML patients coincides with activation of the *LMO2* +1 enhancer, we performed chromatin immunoprecipitation (ChIP)-on-chip analysis in AML patients that expressed high levels of *LMO2*. Acetylation of lysine 9 of histone 3 (H3K9) is a histone mark associated with an open chromatin conformation and is generally observed at promoters and enhancers of active genes. Results from a representative subset of patients revealed a defined peak of H3K9 acetylation corresponding to the *LMO2* +1 enhancer region (Figure 4a; Supplementary Figure 3A) in patients that also showed a broad region of elevated H3K9 acetylation across the HOXA cluster including *HOXA5*, *HOXA7* and *HOXA9* (Figure 4b; Supplementary Figure 3B). Of note, these patients showed high expression of *LMO2*, *HOXA5* and *HOXA9* (Supplementary Figures 2B and 4B). Taken together, these observations are consistent with a model whereby high levels of HOXA proteins in AML cause activation of the *LMO2* +1 enhancer.

### The LMO2 +1 enhancer is active in MLL-ENL immortalized leukaemic cells

Patients with MLL translocations are characterized by upregulation of *HOXA* genes^13, 22^ and can present with myeloid, lymphoid or mixed phenotypes. Of note, interrogation of published gene expression data sets^27^ revealed elevated levels of *LMO2* in AML patients with MLL translocations (Figure 3c). To investigate the possibility of a direct link between MLL-fusion activation of *HOXA* genes and activity of the *LMO2* +1 enhancer in AML leukaemic cells, we took advantage of a mouse cellular model where bone marrow progenitors are immortalized with the fusion protein MLL-ENL.^28^ These cells generate AML *in vivo* when transplanted into lethally irradiated congenic recipients and upregulate expression of *Hox* genes.^29^

**Figure 5:**
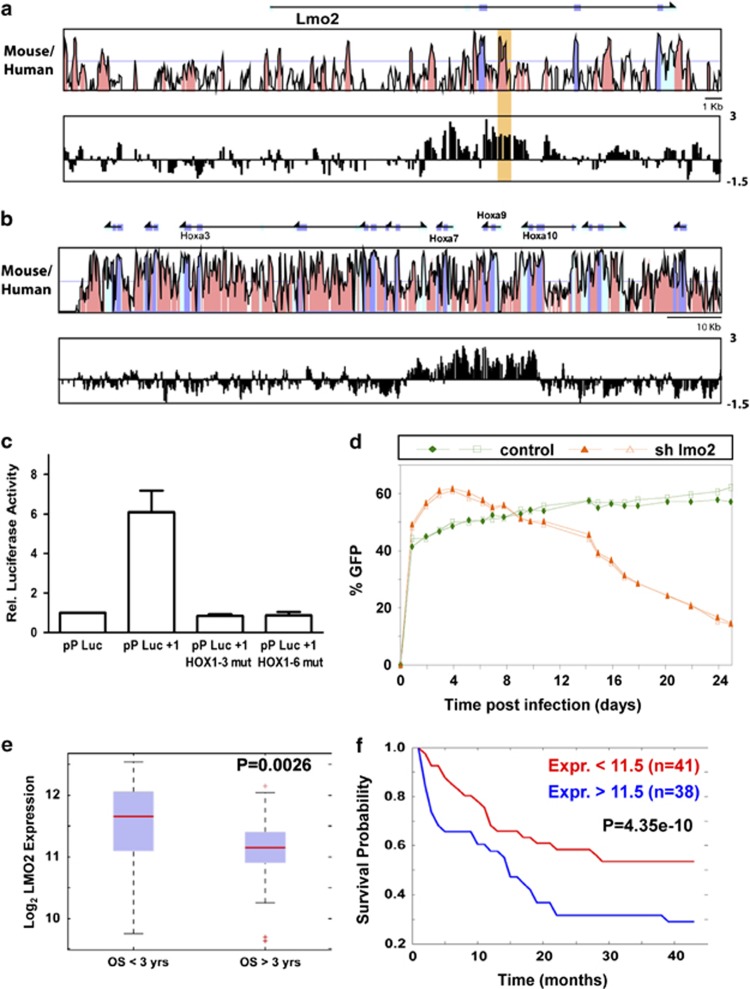
Levels of *Lmo2* control proliferation in MLL-ENL transduced mouse leukaemic cells and correlate with overall survival in AML patients. (**a**) MLL-ENL transduced mouse bone marrow cells show enhanced H3 lysine 9 acetylation at the *Lmo2* +1 similar to primary patient samples. Shown is an MVista representation of mouse/human sequence conservation with +1 enhancer highlighted, with a CHIP-on-chip results shown underneath. Annotations are as in Figure 2a. (**b**) MLL-ENL transduced mouse bone marrow cells show enhanced H3 lysine 9 acetylation across the HoxA cluster similar to primary patient samples. An MVista representation of mouse/human sequence conservation with the ChIP-on-chip results underneath is shown. Annotations are as in Figure 2a. (**c**) The *LMO2* +1 element functions as a transcriptional enhancer in MLL-ENL transduced bone marrow progenitors. Cells were electroporated with luciferase reporter constructs containing the pP, the promoter together with the wild-type +1 enhancer, or the promoter with two different mutant versions of +1 enhancer. Mean and s.e.m. for at least two independent transfections (each one performed in triplicate) are shown. Values are expressed relative to the vector containing the luciferase gene under the control of the minimal pP alone (pP-Luc). (**d**) Knock-down of *Lmo2* in MLL-ENL transduced cells results in a competitive growth disadvantage. MLL-ENL immortalized bone marrow progenitors were transduced with constructs containing shRNA against *Lmo2* (triangles) or luciferase (squares) as a control. GFP presence was monitored over 25 days after infection and percentages of GFP-positive cells are indicated. The results from a representative experiment performed in duplicate are shown. (**e**) Overall survival over 3 years in a cohort of 79 AML patients shows statistically significant association with levels of *LMO2* expression at diagnosis. Shown is a boxplot of expression levels for patients with overall survival of less (left) and more (right) than 3 years, with the corresponding *P*-value shown at the top right. (**f**) Lower *LMO2* expression at diagnosis shows a strong association with improved survival. Kaplan–Meier survival curves for AML patients with log 2 expression scores for *LMO2* higher than 11.5 (blue curve) or lower than 11.5 (red curve) at diagnosis are shown. Corresponding *P*-value is also shown.

ChIP-on-chip analysis for H3K9 acetylation in these cells (Figures 5a and b) revealed a pattern that was remarkably similar to that previously observed in AML patients, with strong enrichment at the *Lmo2* +1 enhancer region (Figure 5a). As for the patient samples, enrichment over the *Lmo2* +1 enhancer was accompanied by high levels of histone H3 acetylation at the HoxA cluster (Figure 5b). We also performed in these cells ChIP analysis for H3K4 monomethylation, a histone mark associated with active enhancers, and we found high levels of this histone modification at the *Lmo2* +1 region (Supplementary Figure 5) thus confirming enhancer characteristics for this element. Furthermore, transfection experiments in these immortalized cells with constructs that contained the luciferase gene under control of the *LMO2* pP with and without the +1 enhancer showed a specific increase in luciferase activity in the presence of the enhancer, which was completely abolished following simultaneous mutation of the first three or all six HOX binding sites present in the *LMO2* +1 enhancer (Figure 5c). Taken together, these experiments demonstrate that the *LMO2* +1 enhancer is active in MLL-ENL immortalized myeloid leukaemic cells, and that this activity is dependent on homeobox consensus binding sites.

### Lmo2 knock-down impairs growth of MLL-ENL transduced progenitors but does not abrogate leukaemic potential

We next explored potential roles for *Lmo2* in controlling proliferation of MLL-ENL transduced cells as well as their ability to cause leukaemia following transplantation into irradiated recipients. To this end, we transduced MLL-ENL immortalized progenitors with a retrovirus expressing shRNA previously shown to knock down the expression of mouse *Lmo2*. Transduced cells express GFP as well as the shRNA of interest so that transduced cells can be monitored during extended culturing. Cells transduced with the *Lmo2* knock-down construct showed reduced expression of *Lmo2* (Supplementary Figure 6A) and were outgrown by untransduced cells after 25 days (Figure 5d). Importantly, no such phenotype was observed when cells were transduced with either empty vector or a negative control knock-down construct. To confirm cell specificity of the observed phenotype, we repeated the *Lmo2* knock-down assays in a non-leukaemogenic haematopoietic progenitor cell line (HPC-7) that expresses high levels of *Lmo2*. In this case no effect could be detected (Supplementary Figure 6B) even though *Lmo2* expression was reduced to <25% of the level seen in controls (Supplementary Figure 6C). We also performed similar experiments in the human cell line U937 that carries the CALM-AF10 translocation where the importance of HOXA5 has been shown^23^ (Supplementary Figure 7A). Similarly to the MLL-ENL cells, U937 transduced with a specific human *LMO2* knock-down construct was outgrown by untransduced cells. We also performed ChIP for H3K9 acetylation and H3K4 monomethylation and transfection experiments in these cells, which demonstrated that the *LMO2* +1 enhancer is active in U937 myeloid cells, and that its activity is dependent on homeobox consensus binding sites (Supplementary Figures 7B and C).

Lethally irradiated mice transplanted with MLL-ENL immortalized bone marrow progenitors develop AML following a latency period of an average of 90–100 days, with leukaemic cells expressing the myeloid surface markers Mac-1 and Gr-1 as well as *Lmo2*.^28^ We hypothesized that maintenance of high *Lmo2* levels could be important for the ability of these cells to cause AML *in vivo*. We therefore transduced MLL-ENL immortalized progenitors with retrovirus expressing shRNA against *Lmo2* and, following selection in puromycin, cells were transplantated into lethally irradiated mice using co-transplanted normal bone marrow cells as radioprotectant. No significant differences could be observed in the latency period when *Lmo2* was knocked down compared with controls (data not shown). Reduction in expression of *Lmo2* was confirmed by quantitative real-time PCR before transplantation and in bone marrow cells obtained from leukaemic mice (Supplementary Figure 8). Our experiments therefore suggest that reducing levels of *Lmo2* expression impair the growth of MLL-ENL immortalized progenitors when cultured *in vitro*, but that these effects are offset *in vivo*, where the disease latency is similar.

### Levels of *LMO2* expression at diagnosis can impact on patient outcome in cytogenetically normal AML

To further explore potential consequences of elevated *LMO2* expression levels in AML, we interrogated published gene expression profiling data sets, specifically focusing on the cytogenetically normal subset of AML patients, because there is an unmet need to develop better prognostic markers within this biologically heterogeneous group. Of note, overall survival for >3 years in a cohort of 79 cytogenetically normal AML patients^30^ correlated specifically with lower levels of *LMO2* at diagnosis (*P*=0.0026; see Figure 5e). Moreover, when we partitioned all patients based on high or low *LMO2* expression (log2 expression score higher than 11.5 (*n*=38) or lower than 11.5 (*n*=41)), a clear correlation between patient outcome and levels of *LMO2* at diagnosis was noted (*P*=4.35e−10; see Figure 5f). Similar results were validated in an independent study of 134 patients (Supplementary Figure 9). Taken together, therefore, the data presented here suggest that potential roles for *LMO2* in leukaemia may extend beyond its traditional function as a T-ALL oncogene, and at least in some patients involve activation of an early developmental HOX-LMO2 regulatory hierarchy.

## Discussion

### The *LMO2* +1 enhancer as a candidate target of HOX activity in early development

LMO2 has thus far been studied predominantly in the context of normal and malignant haematopoiesis. Here, we report comprehensive *in vivo* transgenic analysis of an *LMO2* enhancer driving non-haematopoietic expression. When analysed in detail, the non-haematopoietic expression pattern defined by the +1 enhancer was very similar to that of the *Lmo2* knock-in transgenic animals.^16^ *Lmo2* expression in the posterior tail region is conserved in other species such as Xenopus,^31^ and was adjacent to a region known to contain multipotent neuromesodermal progenitors until about day E12.5 of embryonic development.^32^ Activity of the *LMO2* +1 enhancer in limb and tail bud regions depended on Hox and E-box motifs, and was anatomically consistent with known expression domains of HoxD and HoxA family members as well as bHLH transcription factors.

Few downstream targets for either HOX or bHLH factors are known at early developmental stages. Our results raise the possibility that *LMO2* may be an important functional mediator of HOX and/or bHLH function. E9.5 LMO2^−/−^ embryos were reported to be shorter than their wild-type littermates, consistent with a role in posterior growth^2^ and a *Drosophila* homologue of *LMO2* is involved in dorsal-ventral boundary and wing patterning,^33^ suggesting an evolutionarily conserved function of LMO proteins in patterning. However, *Lmo2* knockout embryos die by E10.5^2^ thus complicating analysis of *Lmo2* function in tail bud extension and patterning. Taken together, our analysis of *LMO2* +1 enhancer function during mouse embryogenesis establishes a previously unknown HOX/LMO2 hierarchy, which may represent a useful starting point for the wider dissection of regulatory networks controlling early mesodermal/axial development.

### An HOXA-LMO2 hierarchy is operational in a subset of acute leukaemia patients

T-ALL is characterized by a number of distinct chromosomal abnormalities as well as acquired mutations.^34^ Importantly, these disparate initiating lesions cause similar disease phenotypes, suggesting convergence on shared downstream leukaemogenic pathways. Here, we propose one such shared pathway, the HOX-LMO2 axis, mediated via the *LMO2* +1 enhancer. The HOXA factor with transactivation potential in our experiments was HOXA5, which is expressed at high levels in the presence of MLL translocations^12, 35^ and has recently been shown to be critical for the leukaemic phenotype in a mouse model of CALM-AF10 driven T-ALL.^23^ Our data are consistent with a direct path from elevated *HOXA* levels to ectopic expression of *LMO2*, and therefore provide potential mechanistic insights into the dysregulation of transcriptional programmes in a larger fraction of T-ALL patients. We also demonstrate that the *LMO2* +1 enhancer is activated in AML samples with elevated *HOX* expression, both in mouse models and in primary patient samples. Information on functionally relevant downstream mediators of HOX factors in AML has remained surprisingly sparse although this may change in the near future given the recent reports of genome-wide mapping of HOXA9 binding sites in a mouse model of AML.^36^

We obtained contrasting results following knock-down of *Lmo2* in *in vitro* and *in vivo* experiments. In cell culture under cytokine-mediated self-renewal conditions, high *Lmo2* levels were associated with a proliferative advantage, and therefore correlated with the hyperproliferation/self-renewal phenotype reported by McCormack *et al.*^37^ in T-cell progenitors ectopically expressing high levels of *Lmo2*. Reducing *Lmo2* levels did not however affect latency of leukaemia onset in an adoptive transfer setting, suggesting that elevated *Lmo2* levels may be dispensable, or that other mutations may compensate for their loss in the context of this particular assay. However, four observations reinforce a potential role for elevated *LMO2* expression in leukaemias other than T-ALL. First, we have recently reported *LMO2* upregulation in the evolution of leukaemia stem cell activity and disease generation in a mouse model of AML.^38^ Second, retroviral integration screens for the identification of new contributors to leukaemia development have reported integration into the murine *LMO2* locus not only in T-ALL but also in B-ALL and AML (RTCGD database), with all translocations occurring 5′ of the *LMO2* coding region and thus presumably enhancing its expression. Third, retroviral overexpression of *LMO2* in Arf^−/−^ thymocytes was recently reported to cause AML in mouse transplant models.^39^ Finally, we now report a correlation of high LMO2 levels at diagnosis with patient outcome (this study). Given that LMO2 is able to confer self-renewal ability to thymic progenitors,^37^ it is attractive to speculate that similar processes may have a role in AML, which would be consistent both with the growth phenotype observed in our knock-down studies and with the relatively poor outcome associated with patients expressing high levels of *LMO2* (Figure 5f).

### Aberrant resurrection of an embryonic regulatory hierarchy in leukaemia cells

Similarities between cancers and embryonic tissues were first reported nearly 200 years ago and gave rise to the so-called ‘embryonal rest' theory of cancer,^40^ although contribution of embryonic stem cells to cancer is now largely discounted with the exception of specific cancers such as teratocarcinoma. Tissue stem/progenitor cells have more recently emerged as likely targets for malignant transformation in a range of leukaemias as well as solid cancers.^41^ Moreover, cancers themselves often maintain a differentiation hierarchy and are maintained by cancer stem cells.^42^

Ectopic *LMO2* expression in T-cell progenitors confers stem cell-like properties giving rise to serially transplantable cells with self-renewal properties.^37^ These cells are thought to act as a reservoir for the acquisition of additional mutagenic lesions that will ultimately result in transformation to acute leukaemia. Malignant transformation is therefore associated with an early acquisition of tissue stem cell characteristics. Our analysis of the *LMO2* +1 enhancer now shows that leukaemic cells can exploit regulatory hierarchies resurrected from normal embryonic development, and therefore provides molecular evidence for parallels between early embryonic cells and cancer. Further analysis of this redeployment of an embryonic gene regulatory control mechanism not only revealed a previously unknown mechanism underlying ectopic expression of *LMO2*, but also suggested that elevated *LMO2* levels may contribute to the disease phenotype in AML. Finally, we would speculate that similar phenomena may occur in other cancers, in that malignant cells will redeploy components of regulatory networks outside of their normal context if this provides a growth advantage.

## Materials and methods

### Custom arrays and ChIP-on-chip assays

Probes spanning the human *LMO2* and *HOXA* loci (build hg18; chr11:33834370-33871997 and chr7: 27091185-27213139, respectively) and mouse *Lmo2* and *HoxA* loci (build mm8; chr2: 103746756-103785459 and chr6: 52076700-52196929, respectively) were generated using eArray software (Agilent Technologies, Wokingham, UK). Microarrays were printed using Agilent's SurePrint technology and ChIP-chip assays were performed as described.^8^

### Patient samples

Peripheral blood samples were collected from patients with AML showing ⩾80% blasts. Informed consent was obtained in accordance with the declaration of Helsinki and local ethical guidelines. Mononuclear fraction was obtained by initial dilution 1:1 in phosphate-buffered saline, followed by density gradient centrifugation using Histopaque (Sigma-Aldrich, Dorset, UK). Cytogenetic information can be found in Supplementary Figures 2 and 4A.

### Transfection assays

MLL-ENL immortalized cells were transiently transfected and assayed as described.^43^ For transactivation assays, 293T cells were transfected with 1 μg *luciferase* construct in combination with 3 μg of either pcDNA3HOXA5 or pcDNA3HOXA7 (generous gift from Dr R Polakowska, La Celle *et al.*^44^) or pcDNA3HOXA9 (generous gift from Dr NR Yaseen, Washington University) or the empty vector pcDNA3 as control using ProFection (Promega, Southampton, UK). Each transfection and transactivation was performed on at least 2 different days in triplicate. HOXA5 cDNA was obtained by PCR, subsequently cloned in pcDNA3 and confirmed by sequencing.

### Retroviral production and transduction

MLL-ENL cDNA subcloned into the pMSCV/IRES-*Neomycin* was kindly provided by A Warren (Cambridge). Retroviral transduction of bone marrow cells, serial replating and transplantation assays were performed as described.^45^ Factor-dependent cells were generated by serial replating and maintained in liquid medium supplemented with 6 ng/ml recombinant interleukin 3 (Peprotech, London, UK). shRNA plasmid against *Lmo2* was a gift from Gerd Blobel (University of Pennsylvania, Tripic *et al.*^46^). Retrovirus production was carried out using the pCL-Eco Retrovirus Packaging Vector (Imgenex, San Diego, CA, USA). MLL-ENL inmmortalized cells were infected with retrovirus by centrifugation using 4 μg/ml polybrene (Sigma-Aldrich) and maintained in media supplemented with IL3.

### Reporter constructs and transgenic assays

Human enhancer sequences were used for reporter constructs. Detailed information is available on request. Transgenic embryos were produced by pronuclear injection, and analysed as previously described.^8^ All animal experiments were performed in accordance with United Kingdom Home Office rules and were approved by Home Office inspectors.

### Flow cytometry and competitive proliferation assay

GFP fluorescence analysis was performed using a FacsCalibur analyser (BD, Oxford, UK). Dead cells were excluded using the 7-aminoactinomycin (7AAD) stain. Competitive proliferation assays in liquid culture were performed by monitoring the GFP-positive cell-fraction over a 25-day time course.

### Bioinformatic analysis

Expression data for 72 T-ALL patients classified by the major molecular cytogenetic/expression signatures were downloaded from GEO (GSE10609^14^). Expression data for 79 cytogenetically normal AML patients were downloaded from GEO (GSE12417^30^). Expression data from human cord blood CD34^+^ cells, transformed with MLL-AF9 (*n*=9) and AML1-ETO (*n*=6), were downloaded from GEO (GSE7011^27^). Heatmaps, boxplots and Kaplan–Meier survival curves were generated using MATLAB software (MathWorks, Cambridge, UK). Associated *P*-values were calculated using Kolmogorov–Smirnov test.
