# Supplementary material for: HOX-mediated LMO2 expression in embryonic mesoderm is recapitulated in acute leukaemias
Source: Oncogene. 2013 May 27;32(48):5471–80. doi: 10.1038/onc.2013.175 (PMC3898495; doi:10.1038/onc.2013.175)
Supplement: Supplementary Information [file onc2013175x1.pdf]

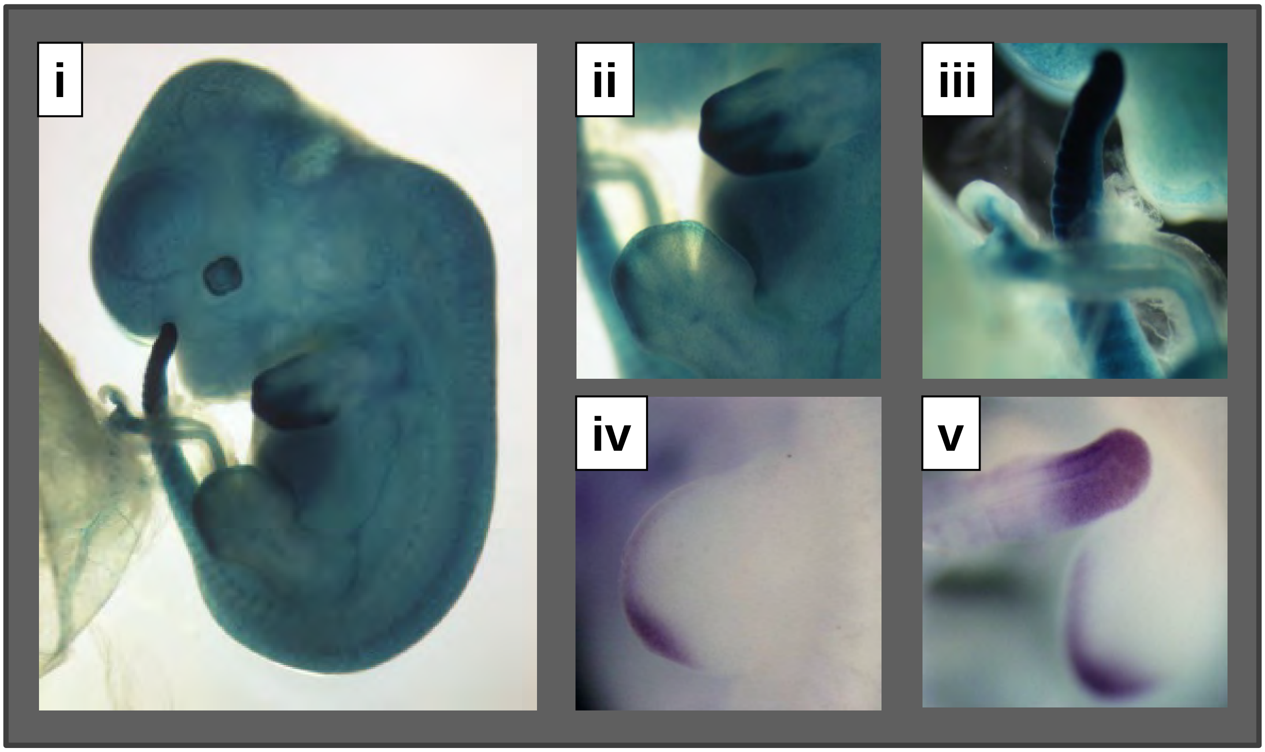

Supplementary Figure 1

A

Patient	Karyotype
**Patient 1**	46,XX[20]
**Patient 2**	5q-, 7q-, 7-, +8 [20]
**Patient 3**	46,XX,del(3)(?p11p21)[14]/46,XX[17]
**Patient 4**	46,XX[20]

B

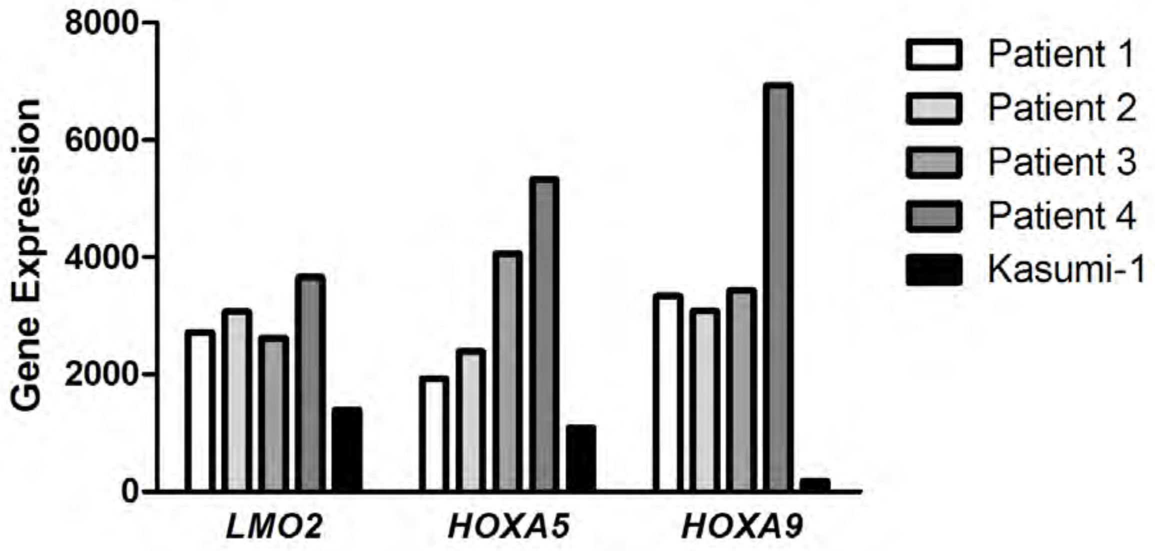

Supplementary Figure 2

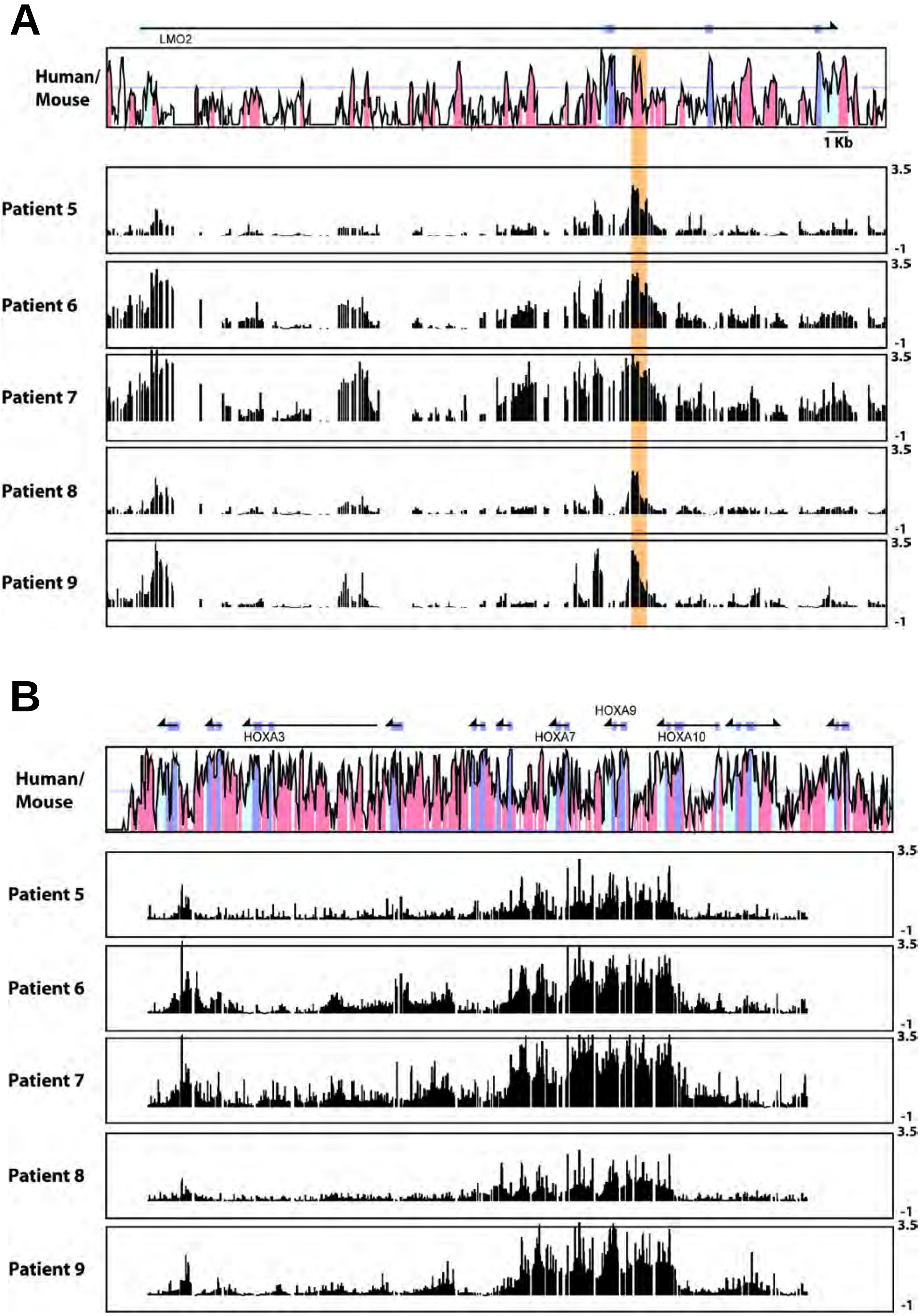

Supplementary Figure 3

A

Patient	Karyotype
**Patient 5**	46,XX[20]
**Patient 6**	46,XY,add(3)(q?26)[6]/47,XY,add(3)(q?26),+mar[2]/47,XY,+mar[3]
**Patient 7**	46,XY,t(3;7)(q26;q34)[3]
**Patient 8**	46,XX[25]
**Patient 9**	46,XX[20]

B

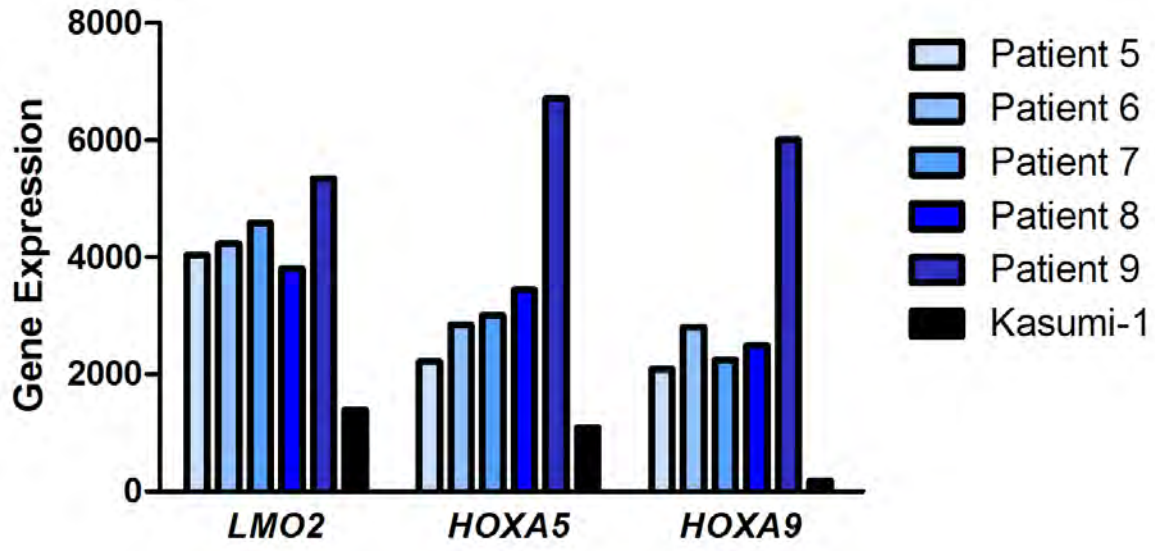

Supplementary Figure 4

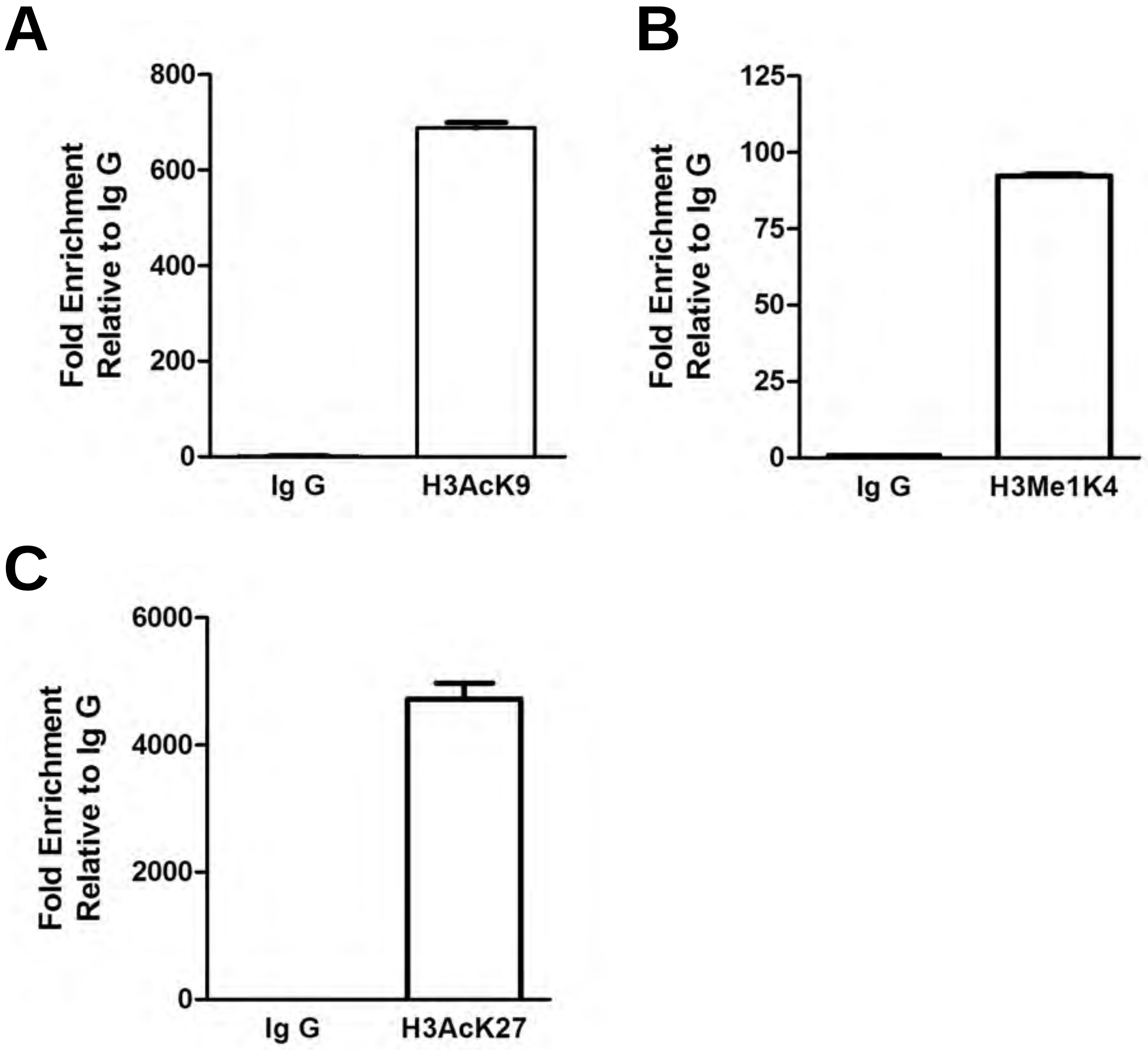

Supplementary Figure 5

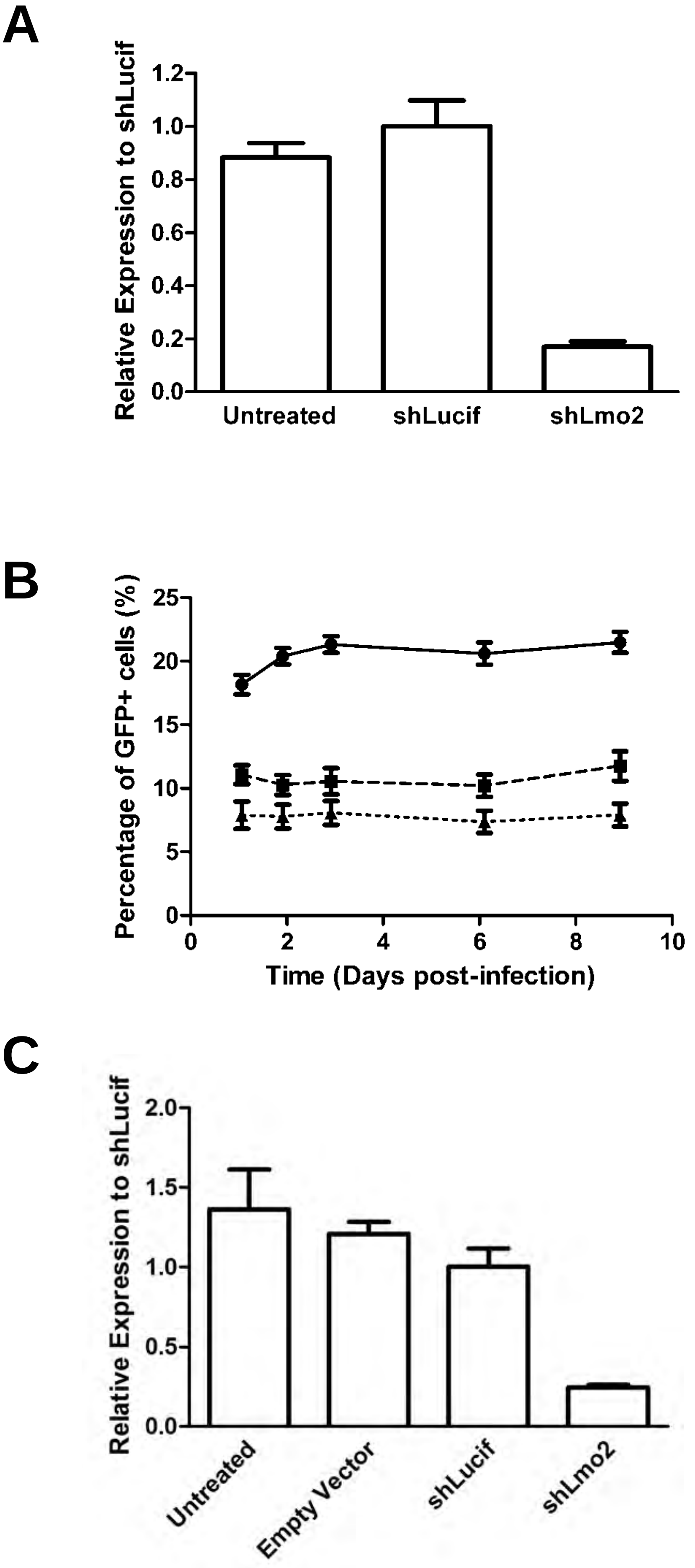

Supplementary Figure 6

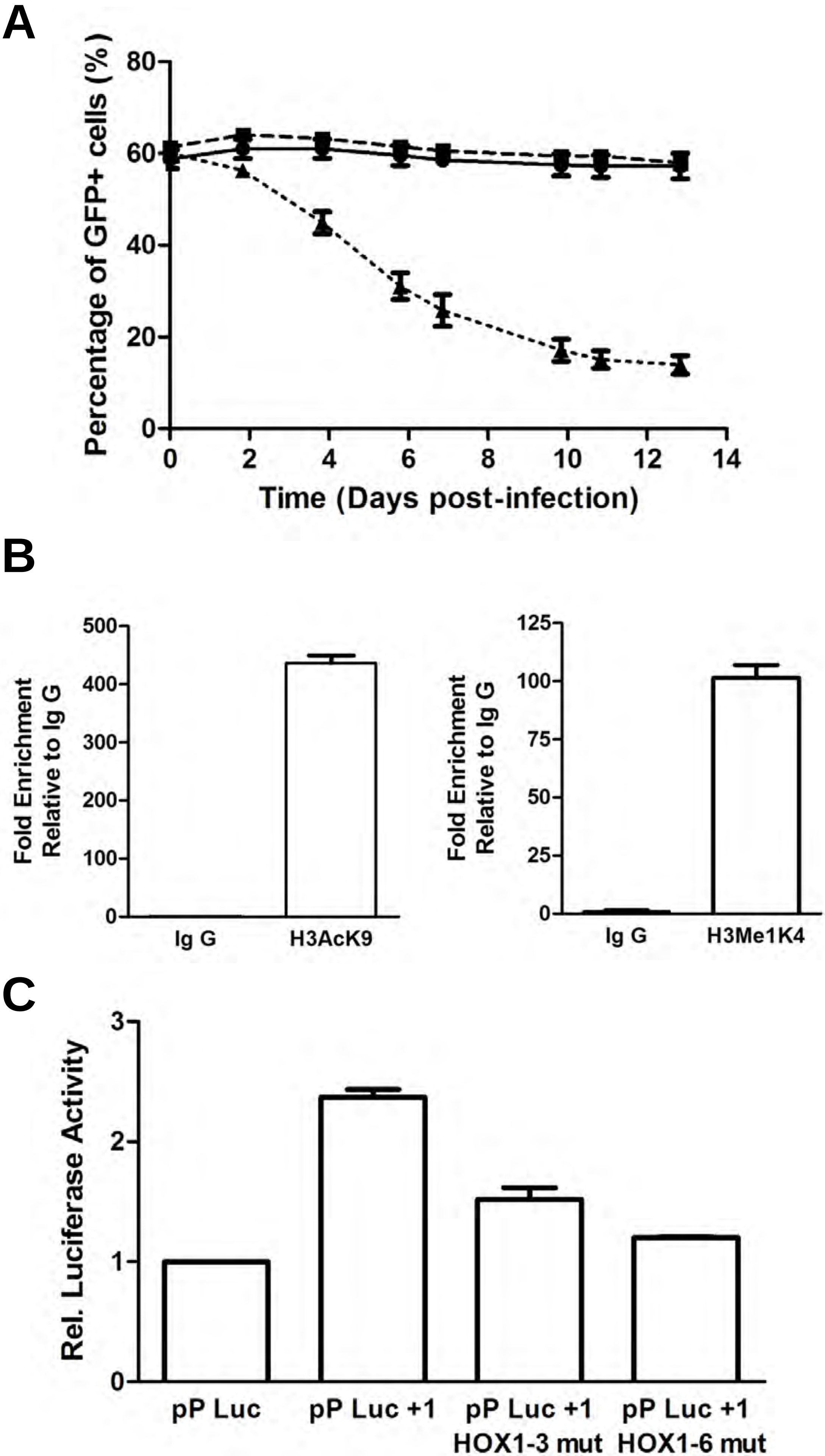

Supplementary Figure 7

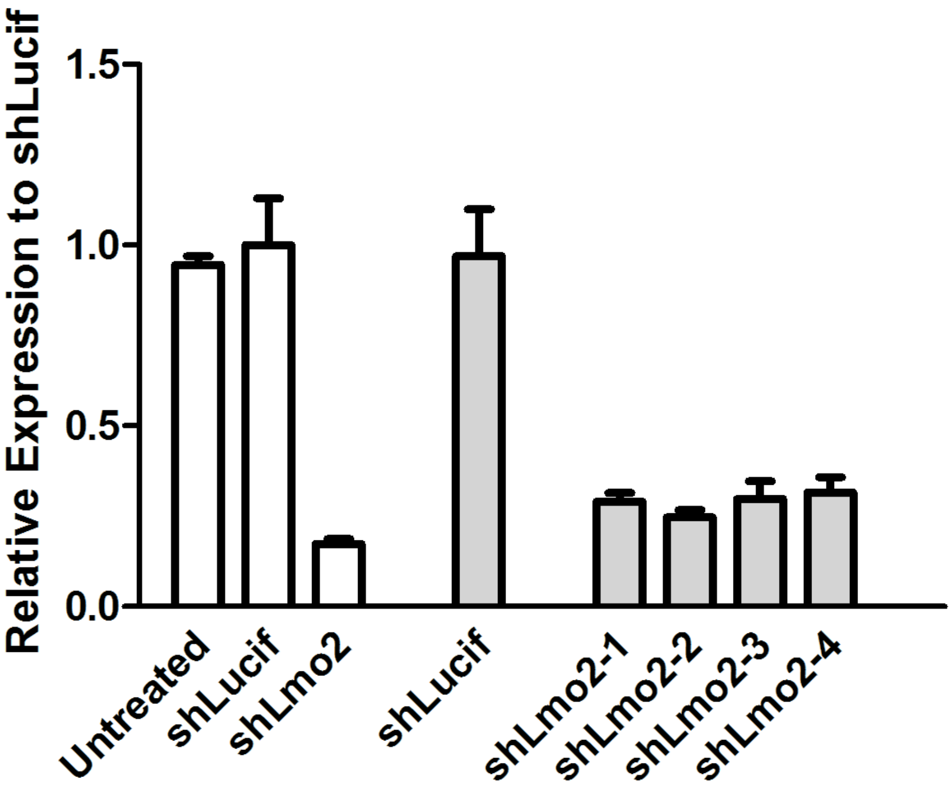

Supplementary Figure 8

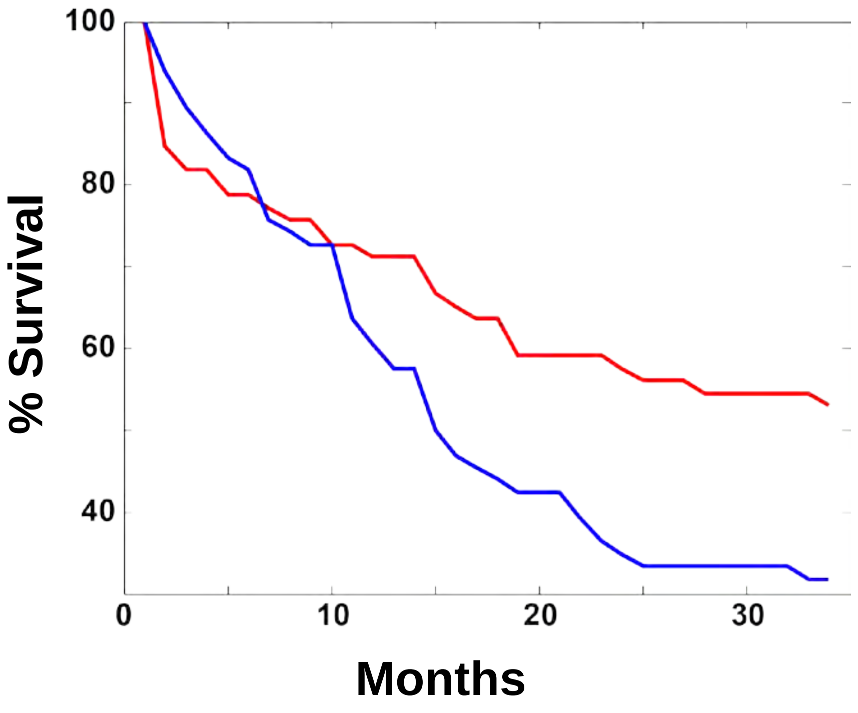

Supplementary Figure 9

Supplementary Figure 1: Non-haematopoietic expression domains of LMO2 expression during mouse embryonic development. Representative mouse embryo at E12.5 (i) showing X-Gal reporter expression in a *Lmo2*$^{+/LacZ}$ knock-in, with close-up views of staining in developing limbs (ii) and tail bud (iii) shown to the right. The original *Lmo2–LacZ* fusion gene targeting vector was generated by introducing a *LacZ-neo* construct in the 24th codon of *Lmo2* (exon 2) where it was linked to the 2nd codon of *lacZ* by a 12-bp linker sequence {Yamada, 2000 #34}. Panels (iv) and (v) confirm endogenous expression by whole-mount in situ hybridisation at embryonic day E10.5 (from Embrys website - http://embrys.jp/ -).

Supplementary Figure 2: Patient information. A) Table shows karyotype corresponding to patients in Figure 4. **B)** Normalized gene expression data for *LMO2*, *HOXA5* and *HOXA9* in patients in Figure 4 and Kasumi-1 cells, which carries the leukaemia fusion genes MLL-AF9.

Supplementary Figure 3: Acetylation of histone H3 lysine 9 indicates that the *LMO2* +1 enhancer is active in primary AML patient samples showing activation of the *HOXA* cluster. A) ChIP-on-chip analysis of the human *LMO2* locus in five additional patient samples shows a peak of H3 lysine 9 acetylation at the *LMO2* +1 enhancer. MVista representation of human/mouse sequence conservation is shown at the top with the +1 enhancer highlighted. Annotations are as in Figure 2A. **B)** ChIP-on-chip analysis of the *HOXA* cluster shows elevated H3 lysine 9 acetylation across central/posterior HOXA genes in the same five patient samples. Enrichment values are calculated as fold enrichment over the mean intensity across the whole locus and expressed as log base 2. Cytogenetic and expression information for these patients can be found in Supplementary Figure 4.

Supplementary Figure 4: Patient information. A) Table shows karyotype corresponding to patients in Supplementary Figure 3. **B)** Normalized gene expression data for *LMO2*, *HOXA5* and *HOXA9* in patients in Supplementary Figure 3 and Kasumi-1 cells, which carries the leukaemia fusion genes MLL-AF9.

Supplementary Figure 5: The LMO2 +1 enhancer carries epigenetic chromatin marks typical of enhancers in MLL- ENL immortalised progenitors. Real-time PCR analysis of ChIP assays in MLL- ENL immortalised progenitors cells performed with antibodies against H3 lysine 9 acetylation (A), H3 lysine 4 monomethylation (B) and H3 lysine 27 acetylation (C) at the *Lmo2* +1 enhancer show elevated enrichment at this region. Levels of enrichment were normalized to Ig G. Error bars indicate standard deviation.

Supplementary Figure 6: Effect of *Lmo2* knock down in MLL-ENL immortalised progenitors and HPC7 cells. A) *Lmo2* expression in shLMO2 transduced MLL-ENL immortalised cells. MLL-ENL immortalised bone marrow progenitor cells were transduced with constructs containing shRNA against LMO2 or luciferase. Transduced cells were selected in the presence of 1µg/ml of puromycin and purity (>98%) was checked by FACS analysis. Also expression of untreated MLL-ENL immortalised bone marrow progenitor cells is shown for comparison. Expression of *Lmo2* was measured by real-time PCR. *mHprt* gene expression was used to normalise expression of each population. Values are expressed relative to the expression of the shluciferase population. Error bars indicate standard deviation. **B)** Knock-down of *Lmo2* in HPC-7 cells does not result in a competitive growth disadvantage. HPC-7 cells were transduced with constructs containing shRNA against *Lmo2* (squares), luciferase (triangles) or the empty vector (circles). GFP presence was monitored over 9 days after infection and percentages of GFP positive cells are indicated. Shown are the results from a representative experiment performed in duplicate. **C)** *Lmo2* expression in shLMO2 transduced HPC7 cells. HPC7 cells were transduced with constructs containing shRNA against *Lmo2*, luciferase or empty vector. Transduced cells were selected in the presence of 1µg/ml of puromycin and purity (>98%) was checked by FACS analysis. Also expression of untreated HPC7 cells is shown for comparison. Expression of *Lmo2* was measured by real-time PCR. *mHprt* gene expression was used to normalise expression of each population. Values are expressed relative to the expression of the shluciferase population. Error bars indicate standard deviation.

Supplementary Figure 7: *LMO2* is important for proliferation in U937 cells where the *LMO2* +1 enhancer carries epigenetic marks typical of enhancers and homeobox motifs are important for its activity. A) Knock-down of *LMO2* in U937 cells results in a competitive growth disadvantage. U937 cells were transduced with constructs containing shRNA against *LMO2* (triangles), luciferase (squares) or the empty vector (circles) as a control. GFP presence was monitored over 13 days after infection and percentages of GFP positive cells are indicated. Shown are the results from a representative experiment performed in duplicate. **B)** The *LMO2* +1 element presents the chromatin marks of a transcriptional enhancer in U937 cells. Real-time PCR analysis of ChIP assays in U937 cells performed with antibodies against H3 lysine 9 acetylation and H3 lysine 4 monomethylation at the *LMO2* +1 element show elevated enrichment at this region. Levels of enrichment were normalized to Ig G. Error bars indicate standard deviation. **C)** Homeoboxes present in the *LMO2* +1 element are essential for activity of this element in U937 cells. Cells were electroporated with luciferase reporter constructs containing either the proximal promoter (pP), the promoter together with the wild type +1 enhancer, or the promoter with 2 different mutant versions of +1 enhancer. Mean and SEM for at least two independent transfections (each one performed in triplicate) are shown. Values are expressed relative to the vector containing the luciferase gene under the control of the minimal proximal promoter alone (pP-Luc).

Supplementary Figure 8: Reduction of expression of *Lmo2* can be confirmed before and after transplantation of MLL-ENL immortalised progenitors in mice. MLL-ENL immortalised progenitors were transduced with retrovirus expressing shRNA against *Lmo2* or luciferase. Transduced cells also express GFP. Following selection in puromycin, cells were transplantated into lethally irradiated mice using co-transplanted normal bone marrow cells as radioprotectant. Reduction of expression of *Lmo2* can be confirmed by quantitative real-time PCR in MLL-ENL immortalised progenitors before transplantation (white data series) and in bone marrow cells obtained from leukaemic mice after transplantation (grey data series) with cells transduced with retrovirus expressing shRNA against *Lmo2* (four) or luciferase (one). In all shLmo2 cases, GFP+ cells comprised more than 70% of total cellularity of bone marrow.

Expression of untreated MLL-ENL immortalised bone marrow progenitor cells is also shown for comparison. *mHprt* gene expression was used to normalise expression of each population. Values are expressed relative to the expression of the shluciferase population before transplantation. Error bars indicate standard deviation.

Supplementary Figure 9: Lower *LMO2* expression at diagnosis shows a strong association with improved survival. Gaidzik et al. (Gaidzik et al, 2011) reported gene expression profiles for over 200 AML patients at diagnosis together with clinical data on survival, as part of the German Austrian AML Study Group (AMLSG) [AMLSG trial AML HD98A (ClinicalTrials.gov Identifier: NCT00146120)]. Top 1/3 and bottom 1/3 *LMO2* expressing samples for each subgroup with more than 30 patients were pooled to generate a Kaplan-Meier plot. Blue (n=67) and red (n=67) lines indicate samples with high and low levels of *LMO2* at diagnosis, respectively. Association of low levels of *LMO2* with an overall survival benefit can be seen (P-value= 6.63e-6, calculated using Kolmogorov-Smirnov test).

Supplementary Reference:

Gaidzik VI, Bullinger L, Schlenk RF, Zimmermann AS, Rock J, Paschka P, Corbacioglu A, Krauter J, Schlegelberger B, Ganser A, Spath D, Kundgen A, Schmidt-Wolf IG, Gotze K, Nachbaur D, Pfreundschuh M, Horst HA, Dohner H, Dohner K (2011) RUNX1 mutations in acute myeloid leukemia: results from a comprehensive genetic and clinical analysis from the AML study group. *J Clin Oncol* **29**(10)**:** 1364-1372
